# Dynamic morphology and cytoskeletal protein changes during spontaneous inside-out vesiculation of red blood cell membranes

**DOI:** 10.1007/s00424-014-1483-5

**Published:** 2014-03-12

**Authors:** Teresa Tiffert, Virgilio L. Lew

**Affiliations:** Physiological Laboratory, Department of Physiology, Development and Neuroscience, University of Cambridge, Downing Street, Cambridge, CB2 3EG UK

**Keywords:** Erythrocyte, Erythrocyte membrane, Erythrocyte vesicles, Erythrocyte cytoskeleton, Inside-out vesicles, Merozoite egress

## Abstract

Vesicle preparations from cell plasma membranes, red blood cells in particular, are extensively used in transport and enzymic studies and in the fields of drug delivery and drug-transport interactions. Here we investigated the role of spectrin–actin, the main components of the red cell cortical cytoskeleton, in a particular mechanism of vesicle generation found to be relevant to the egress process of *Plasmodium falciparum* merozoites from infected red blood cells. Plasma membranes from red blood cells lysed in ice-cold media of low ionic strength and free of divalent cations spontaneously and rapidly vesiculate upon incubation at 37 °C rendering high yields of inside-out vesicles. We tested the working hypothesis that the dynamic shape transformations resulted from changes in spectrin–actin configuration within a disintegrating cytoskeletal mesh. We showed that cytoskeletal-free membranes behave like a two-dimensional fluid lacking shape control, that spectrin–actin remain attached to vesiculating membranes for as long as spontaneous movement persists, that most of the spectrin–actin detachment occurs terminally at the time of vesicle sealing and that naked membrane patches increasingly appear during vesiculation. These results support the proposed role of spectrin–actin in spontaneous vesiculation. The implications of these results to membrane dynamics and to the mechanism of merozoite egress are discussed.

## Introduction

Plasma membrane vesicle preparations of right-side-out (ROVs) and inside-out (IOVs) orientation are a valuable and widely used tool that has contributed a wealth of information on the identity, kinetics and vectorial properties of plasma membrane transporters and enzymes [12, 21, 26, 31, 32, 46]. As with many such tools in biological research, the experimental protocols yielding vesicular preparations were the result of inspired trial and error experimentation, ideas on the mechanism by which vesicles were formed taking shape subsequently. One of the most widely used IOV preparations was that originally developed by Steck and collaborators for red blood cell (RBC) membranes [4, 5, 7, 38–40, 43, 44]. The essential procedural steps were as follows: RBCs were first lysed in many volumes of an ice-cold, hypo-osmotic, low-ionic strength medium, free of divalent cations and lightly buffered to pH 7.5–7.8; the pelleted membranes, usually referred to as “ghosts” at this stage, were further washed and incubated at low temperatures in such media. Final shearing of such treated membranes through thin needles rendered the desired vesicles. One idea about the mechanism of formation of IOVs and ROVs by this method was based on the observation that the conditions caused progressive loss of spectrin and actin, leaving a naked membrane devoid of cytoskeletal support, essentially a giant liposome [38, 40, 44]. Subsequent shearing through thin needles fragmented the membranes into sealed vesicles through fleeting transitional open states of random inside-out or right-side-out topology.

An important property of the vesicles generated by the classic Steck–Kant procedure [43] was the functional preservation of nearly all native enzymes and transporters of the RBC membrane. The notable exception was the Ca^2+^-activated K^+^ channel of the RBC membrane (IK1, Kcnn4 [3, 19, 45], also known as the Gardos channel [13, 24, 36]). Searching for the stage in the vesicle-preparation protocol at which channel activity was lost, it was discovered that a vesicular preparation with a high yield of IOVs could be generated within 5–10 min of lysis if the membranes were immediately incubated at 37 °C in the lysis medium [26]. The vesicles generated in this way retained Gardos channel activity [2, 12, 26].

The rapid generation of such “one-step” vesicles from lysed RBCs enabled the vesiculation process to be followed and recorded under the microscope on a temperature-controlled stage [25]. The most surprising finding was that one-step vesicle formation was an entirely spontaneous process; each ghost was seen to transform spontaneously into a bunch of linked vesicles within about 5–8 min at 37 °C, a process at variance with the giant liposome transitional stage model. A detailed study of this process by a variety of techniques showed that spontaneously generated IOVs were formed following a specific sequence of highly dynamic membrane shape changes. A first critical finding was that the unique lysis conditions leading to vesiculation prevented the sealing of the lytic hole [25, 27, 28]. At 37 °C, the membrane around the lytic hole rapidly curled outwards forming a toroid around the opening in most, though not all open ghosts. In a variable but substantial fraction of the ghosts, the initial curling was followed by a protrusive buckling of the membrane area opposite the hole leading to rapid and complete eversion of the ghost membrane, forming inside-out ghosts (IOGs) [25]. Analysis of the dynamic morphology of spontaneously vesiculating membranes in electron microscopic serial sections showed that the curls were the main IOV-forming factory, both in IOGs and in the right-side-out ghosts (ROGs), those which had not buckled and everted. We shall refer to the curling–buckling–eversion–vesiculation sequence of IOGs as the CBEV sequence [23]. This particular vesiculation modality has recently acquired distinct significance in malaria-infected RBCs and is briefly described next.

Using high-speed videomicroscopy and epifluorescence, Abkarian and colleagues [1, 8] studied the process of merozoite egress from RBCs infected with the malaria parasite *Plasmodium falciparum* in culture conditions. They discovered that host-cell rupture and merozoite release at the end of the asexual reproduction cycle of the parasite occurred with the host cell membrane curling outwards around the rupture hole, forming a toroid, and then sequentially buckling, everting and vesiculating, confirming the post-egress vesiculated condition of the host cell membrane [14, 16, 17]. Buckling was shown to aid merozoite ejection and dispersal. Eversion, by removing any residual containment to dispersal, may play an important role in vivo, where egress occurs with the infected cells adhered to endothelial cells in the microvasculature [1, 23]. Vesiculation during egress occurs extremely rapidly; the final appearance of the residual host cell membrane is that of a linked bunch of vesicles [17]. The analogies between the CBEV sequences during merozoite egress and during spontaneous vesiculation of IOGs strongly suggest common mechanisms. However, the experimental conditions and kinetics are markedly different, CBEV during egress being completed in iso-osmotic culture media within about 400 ms at 37 °C [1]. These findings opened a new perspective on the study of the CBEV sequence, from a process of biophysical interest in the experimental generation of inside-out plasma membrane vesicles to a process of much wider and profound biological and medical relevance, rendered amenable to study on the experimental model of the spontaneous vesiculation process.

A molecular mechanism was proposed to explain the three major features of the spontaneous vesiculation process [25]: the dynamic shaping of the emerging vesicles driven by the particular geometry of cytoskeletal breakdown, the formation of free membrane edges and the extensive membrane fusion leading to vesicular sealing. The membrane motions responsible for curling, vesicular shaping and the CBEV sequence of IOGs were attributed to the pattern of cytoskeletal disassembly and breakdown, whereas the fusion events were attributed to interactions between integral membrane proteins lining the extensive open membrane edges during intermediate vesiculation stages, acting like membrane zips.

Independent observations suggested the existence of a direct link between the modality of cytoskeletal breakdown and membrane motions during spontaneous vesiculation [20]. Spectrin (bands 1 and 2) and actin (band 7) [11, 41, 42], which make up about 75 % of the cytoskeletal proteins, dissociate from the membrane in conditions of low ionic strength and pH similar to those in which spontaneous vesiculation occurs, and inside-out vesicles from red cell membranes prepared by different procedures were found to be depleted in spectrin and actin; the surface density of fibrillar projections from the inner membrane face, representing mostly spectrin strands [25, 30], was found to be much reduced in spontaneously formed vesicles relative to ghosts and intermediate vesiculation forms. These observations documented before–after conditions but provided no information about the dynamics and time-course of spectrin–actin losses during the intermediate stages of the spontaneous vesiculation process. If the membrane motions during spontaneous vesiculation result from the peculiar dissociation pattern of the spectrin cortex, it is necessary for spectrin to remain retained for as long as movement is detected. The observed depletion of spectrin–actin in the formed vesicles must therefore represent a late, terminal loss rather than the result of gradual dissociation. The work reported here focusses on the time correlation between morphology and spectrin loss and on the macroscopic deformability properties of a spectrin-free red cell membrane.

## Methods

**Fig. 1:**
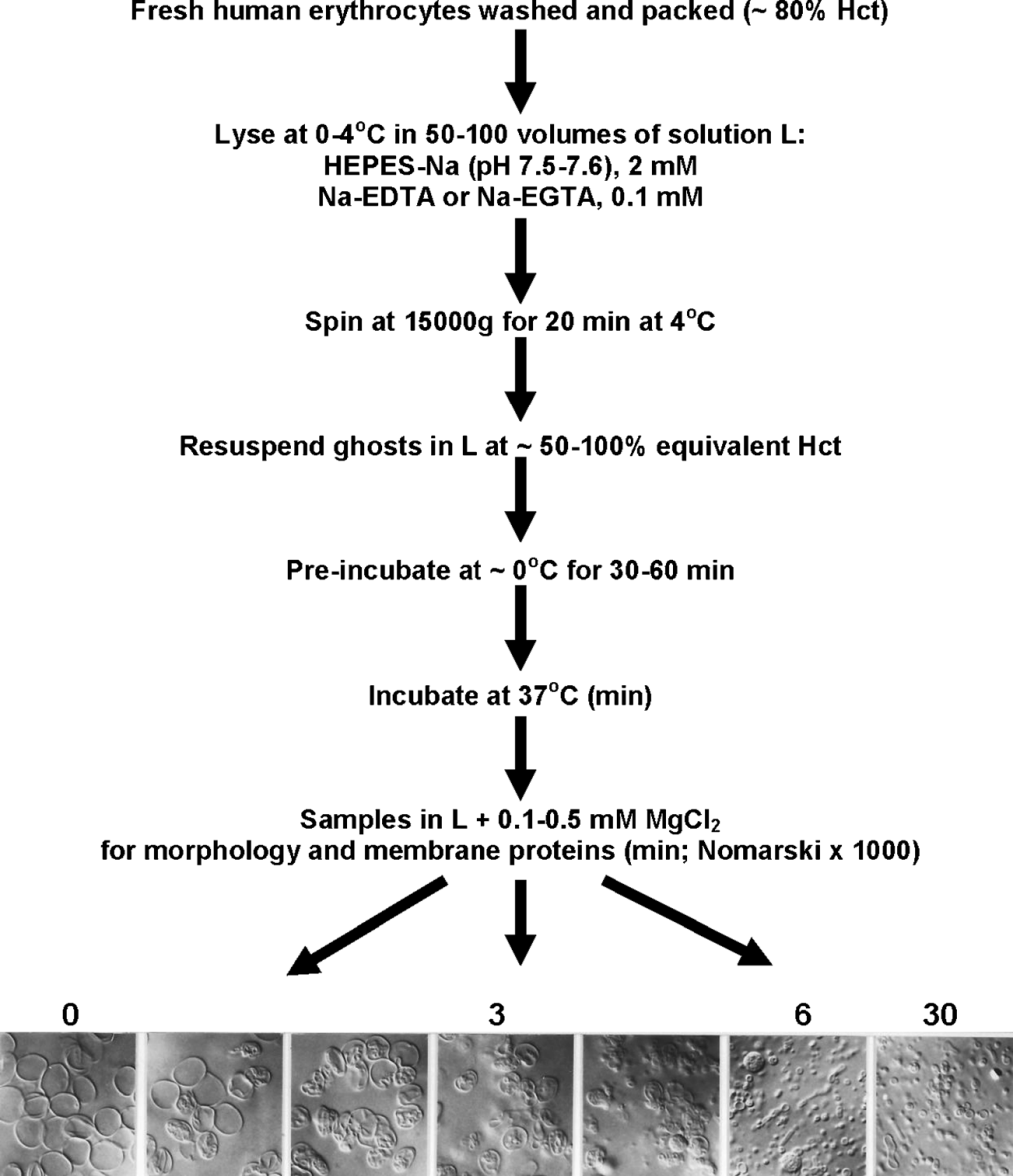
Sequential steps in the experimental protocol for the spontaneous formation of inside-out vesicles. Pre-incubation of the ghost membranes at 0 °C improves vesiculation synchrony in the ghost population during subsequent incubation at 37 °C (see text). The *figure at the bottom* illustrates a sequence of Nomarski views (×1,000) taken from samples at 1, 2, 3, 4, 6 and 30 min of incubation at 37 °C. After the spin (*step 3 from top*), the supernatant was discarded and the pink ghost pellet was gently transferred to a new tube for subsequent resuspension in L (*step 4 from top*), taking care to prevent any contact and mixing with the dark adherent button at the bottom of the centrifuged tube (see “Methods”)

The experimental protocol for the spontaneous formation of IOVs is illustrated in Fig. 1. Briefly, fresh venous blood was obtained from healthy donors after informed consent, using heparinized syringes. Red blood cells were washed three times with 10 vol of a solution containing (in mM) NaCl 142, KCl 3, HEPES-Na (pH 7.5) 10 and EGTA or EDTA 0.1, to chelate extracellular divalent cations. Residual plasma, buffy coat and topmost cell layer were removed after each wash. For vesiculation, the washed red cells were lysed in 50–100 vol of ice-cold solution “L” (in mM): HEPES-Na (pH 7.5) 2.5 and EGTA 0.1. The lysed cells were immediately spun at 15,000×*g* for 15–20 min at 0–5 °C, forming a pink pellet on top of a tiny dark button at the bottom of the centrifuged tube. The supernatant was discarded, and the pink ghost pellet was gently transferred to a new tube avoiding any contact and mixing with the dark adherent button at the bottom of the tube. This button contains residual blood cell contaminants rich in proteases capable of drastically altering the electrophoretic patterns studied here if retained, or imperfectly removed [43]. Conservation of the sodium dodecyl sulphate (SDS)–gel electrophoretic pattern of red cell membrane proteins in the current controls, even after prolonged incubations (see Fig. 5), documents the effectiveness of contaminant protease removal. The transferred ghost pellet was resuspended in ice-cold solution L at an equivalent hematocrit (relative to the original volume of cells) of 50–100 % and the suspension pre-incubated in the ice-bath for 30–60 min to optimize synchronized vesiculation in the ghost population when subsequently incubated at 37 °C. Vesiculation was initiated by transferring the suspension to a water bath at 37 °C. Duplicate 0.1-ml samples of the suspension were taken before and after this transfer at the time intervals indicated in the figures and diluted ten-fold into microfuge tubes containing 0.9 mL of solution L with 0.1–0.5 mM MgCl_2_ to halt further vesiculation [11]. Phase contrast and Nomarski observations and photomicroscopy (Zeiss Photomicroscope III) were performed on these unfixed samples using ×l00 oil immersion objective lenses and temperature-controlled slides.

Samples for SDS–polyacrylamide gradient gel electrophoresis were processed as originally described by Fairbanks and collaborators [11]. The gels were fixed and stained with Coomassie Brilliant Blue. The Mg-arrested samples were ice-cooled and centrifuged at 12,000×*g* for 30 min in a temperature-controlled microfuge at 4 °C (Eppendorff Centrifuge 5402). The membrane pellets and supernatants were separated and independently processed for electrophoresis. The supernatant sample was used to identify the membrane proteins that became fully detached and lost to the medium during the vesiculation process.

## Results

Throughout this investigation, we have made extensive use of four empirical observations that will be documented in more detail in the following sections: (i) Pre-incubation of the lysed RBCs at 0 °C for about 30–60 min immediately after lysis was found to increase the level of synchronization of the vesiculation process among different ghosts relative to non-pre-incubated samples, important for studying the correlation between morphology and membrane protein changes in multi-cell samples of spontaneously vesiculating ghost. The lysis conditions in which IOVs are spontaneously formed (low-ionic strength, divalent cation-free hypotonic media) were shown to prevent sealing of the lytic hole in all ROGs. This opening in all ROGs, as well as the everted condition of the IOGs, ensure free access of the ice-cold lysis medium to the inner membrane surfaces during the pre-incubation period. Synchronization is thus probably the result of a slow coordinated rearrangement of the cytoskeleton to a similar starting configuration in most ghosts; (ii) addition of small concentrations of MgCl_2_ (0.1–0.5 mM) to suspensions of vesiculating ghosts *instantly and reversibly* arrests the vesiculation process. Mg arrest could therefore be used to freeze vesiculating samples in time thus enabling study of time-dependent changes on stabilized, unfixed samples. Other divalent cations (Ca^2+^, Co^2+^) also cause instant vesiculation arrest, but reversibility was only observed after Mg^2+^ washout and re-suspension of the membranes in the divalent cation-free lysis medium. Evidence will be provided that suggests that the vesiculation-arresting effects of divalent cations result from spectrin cross-linking; (iii) to investigate the changes in membrane proteins during IO vesiculation, it was found convenient to retain a trace haemoglobin (Hb) concentration in the medium (5–10 μM) to be used as an indicator of vesicular sealing by its increased membrane association within a newly sealed membrane compartment during sample elution; and (iv) a relatively minor increase in the ionic strength of the lysis and vesiculation medium had no significant effect on the rate of cytoskeletal breakdown, but it dramatically reduced the extent of spontaneous vesiculation leaving a preparation in which most ghosts had the appearance of giant liposomes. This allowed us to explore the physical behaviour of cytoskeleton-free membranes under convective currents.

**Fig. 2:**
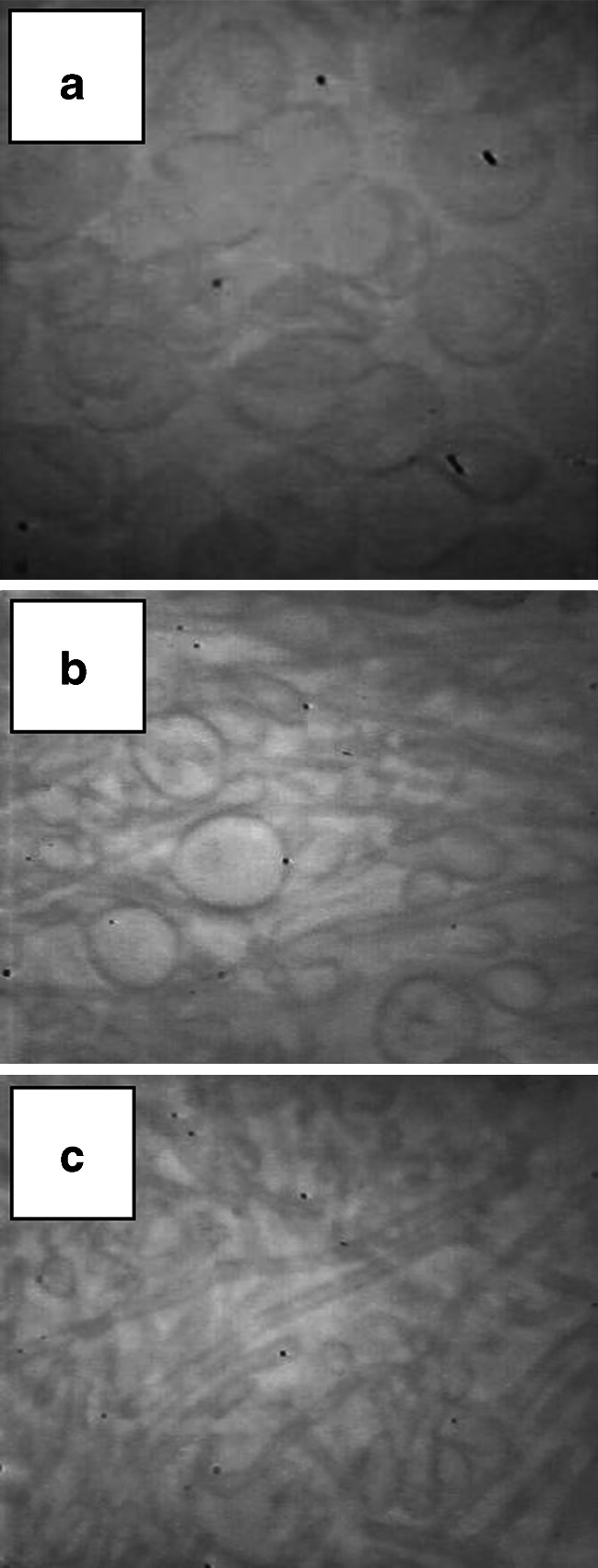
Deformability of cytoskeleton-free red cell membranes under convective currents. **a** Ghosts lysed in solution L and incubated at 37 °C in solution L with added 10 mM NaCl for 30 min. These conditions largely de-couple spectrin–actin loss from spontaneous vesiculation leaving cytoskeleton-depleted, partially vesiculated ghosts. **b**, **c** Effects of mild (**b**) and strong (**c**) tapping of the coverslip on ghost membrane shape changes. The convective currents generated by the tapping were left to subside before the pictures were taken. They illustrate the non-reversibility of the membrane deformations generated by external forces on cytoskeleton-free membranes

In the experiment of Fig. 2, red cells were lysed and pre-incubated in solution L in the cold as per the protocol in Fig. 1 and subsequently re-suspended and incubated at 37 °C in medium L with added NaCl 10 mM. Mg-arrested samples were placed between slide and coverslip and recorded under phase contrast observation. Spontaneous vesiculation was much reduced under these conditions, but ghost appearance changed dramatically as cytoskeletal proteins detached, from an initial stiff, folded body (Fig. 2a) to that of a soft, inflated balloon, easily elongated along minor convective currents (Fig. 2b). Figure 2c, from a 30-min sample, illustrates the effect of gently tapping the coverslip on the appearance of such ghosts under the convective currents generated by this manoeuvre. It can be seen that the membranes deformed into extended tubular structures as would be expected from a two-dimensional liquid devoid of intrinsic shape controls. This simple manoeuvre illustrates the inability of cytoskeleton-free membranes to generate shape changes in the absence of external forces. The spontaneous membrane motions during IO vesiculation must therefore be driven by localized configuration changes within the cortical cytoskeletal mesh as it breaks down. Because these motions take place in the total absence of energy sources other than thermal, they are entropic and must result from potential-to-kinetic energy conversions within the disassembling cytoskeleton, the modality of breakdown of the cortical cytoskeletal mesh shaping the curls and emerging vesicular forms [20]. As argued in the “Introduction”, a necessary condition for this interpretation is that spectrin and actin, the main cytoskeletal mesh components, should be retained for as long as spontaneous motions persist, and we report next the results of experiments designed to test this condition.

**Fig. 3:**
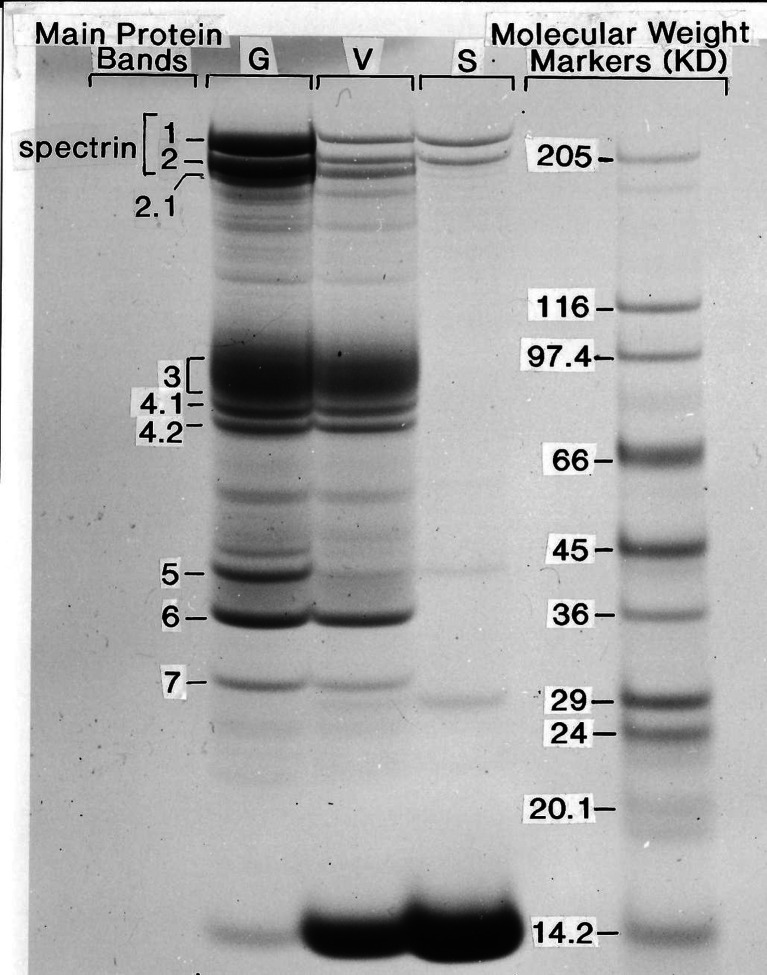
Membrane protein changes during spontaneous inside-out vesiculation. The first there columns (*left* to *right*) show representative SDS–gel electrophoretic patterns of pre-vesiculation ghosts (*G*), post-vesiculation IOVs (*V*) and post-vesiculation supernatants (*S*). The *rightmost column* indicates distances travelled by molecular weight markers on the same gel. The band numbers on the *left-most column* follow the familiar nomenclature established in the pioneering papers of Fairbanks, Steck and collaborators [11, 42]. The most relevant electrophoretic components in the context of the present work are bands 1 and 2 (spectrin), actin (band 7) and monomeric haemoglobin (16 kDa, next to the 14.2-kDa MW marker in this *figure*; the *bottom band* in all membrane protein columns). Because spectrin and actin form the bulk of the cytoskeletal mesh of the red blood cell cortical cytoskeleton, the focus of this investigation is on their time-dependent changes during spontaneous vesiculation. Haemoglobin traces are used to signal the time of vesicular sealing, as explained in “Results”. Although the same volume of sample was deposited on the gels for G and V samples, V samples carried less total protein than G samples because of losses to supernatant. The supernatant column (*S*) identifies the membrane proteins that were fully detached and released to the incubation medium during vesiculation despite their highly diluted state relative to their density in the membrane samples. The monomeric globin band circa 16 kDa can be clearly seen in the V and S samples but not in the open state G samples

Membrane protein changes were followed on pellets and supernatants of vesiculating samples by SDS–polyacrylamide gel electrophoresis (see “Methods”). Figure 3 illustrates the initial (G) and final (V) conditions of membrane proteins in spontaneously vesiculating ghost preparations. The main components of the cytoskeletal mesh are spectrin (bands 1 and 2) and actin (band 5). The spectrin subunits α and β have MWs 220 and 240 kDa, respectively, and the MW of the actin monomers is 43 kDa. The band which corresponds to globin from haemoglobin monomers, the SDS denatured form of the solubilised haemoglobin tetramer, has a MW of about 16 kDa. The results (Fig. 3) document a dramatic loss of spectrin and actin in spontaneously formed IOVs (V) relative to pre-vesiculation ghost membranes (G), a pattern similar to that recorded for IO vesicles prepared by the Steck–Kant method [11, 29]. The small and variable residual amount of spectrin–actin associated with vesiculated membrane pellets can be attributed to asynchronies and heterogeneities in vesiculation patterns: partially vesiculated ghosts, incomplete detachment of spectrin strands visible as isolated residual fibrillar projections in electron microscopic images [20, 25, 30] and detached cytoskeletal proteins retained within sealed vesicles. The reduction in spectrin and actin contrasts with the dramatic increase in membrane-associated haemoglobin (Fig. 3, V relative to G). To establish whether this haemoglobin was bound to or trapped within the vesicles, the vesiculated membranes were exposed to two consecutive freezing–thawing cycles to disrupt permeability barriers. Freezing–thawing prior to SDS–gel electrophoresis fully removed all Hb-membrane association in the vesiculated samples (not shown), indicating that the observed membrane-associated Hb had been trapped within sealed vesicles rendered permeable by freezing and thawing.

**Fig. 4:**
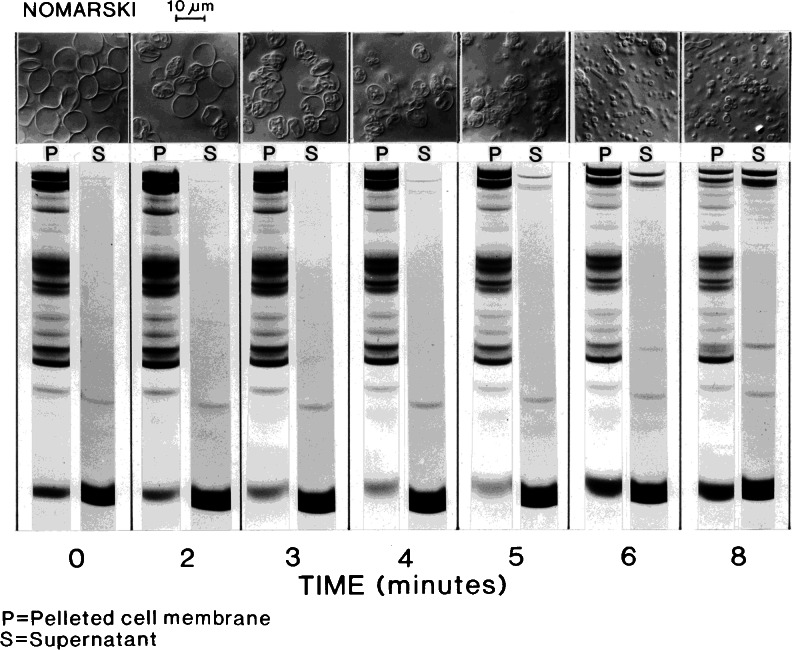
Time relations between morphological and membrane protein changes during spontaneous inside-out vesiculation of red cell membranes. Red cells were lysed and resuspended at 50 % equivalent haematocrit in L at 0 °C. The suspension was kept at 0 °C in an ice-bath. A 5-μL sample was placed between slide and coverslip on a temperature-controlled stage, initially set at 4 °C, of a Zeiss photomicroscope under Nomarski (×1,000) observation. Vesiculation was initiated (*t* = 0) both in the suspension and slide by transfer to a water bath at 37 °C and by rising the stage temperature to 37 °C, respectively. Suspension samples for membrane (*left of paired columns*) and supernatant (*right of paired columns*) proteins were taken at the indicated times and processed as described in “Methods”. Nomarski photo-images were taken at the indicated times and are shown in the figure lined up above the time-corresponding membrane protein columns. Note that the reduction of spectrin from pellets (bands 1 and 2, Fig 3) and the appearance of spectrin in supernatants become detectable at about 5 min, just before large-scale vesicular sealing as indicated by haemoglobin trapping (*bottom band*). Allowing for imperfect synchronization, the results suggest large-scale spectrin retention during the early dynamic stages of the spontaneous vesiculation process

The time-dependent changes in RBC membrane proteins throughout the spontaneous IO vesiculation process were followed in parallel with morphological changes under Nomarski optics observation (Fig. 4). During the first 5 min, we see profound morphological changes along previously described patterns [25] with hardly any detectable change in membrane proteins. The release of spectrin and actin to the supernatant becomes detectable only after the 5-min sample in this series. Spectrin detachment from the membranes to the supernatant is progressively evident in the 6- and 8-min samples, and the weaker actin band is clearly detectable in the 8-min supernatant sample. Between the fifth and sixth minute, there is an abrupt increase in the membrane-associated monomeric Hb band, in parallel with late formation of vesicles. These results clearly show that the main cytoskeletal mesh components, spectrin and actin, remain associated with the cell membrane during the dynamic morphological changes leading to vesiculation and that they only become fully dissociated at the final vesicle sealing stage, when Hb becomes trapped within sealed vesicles.

**Fig. 5:**
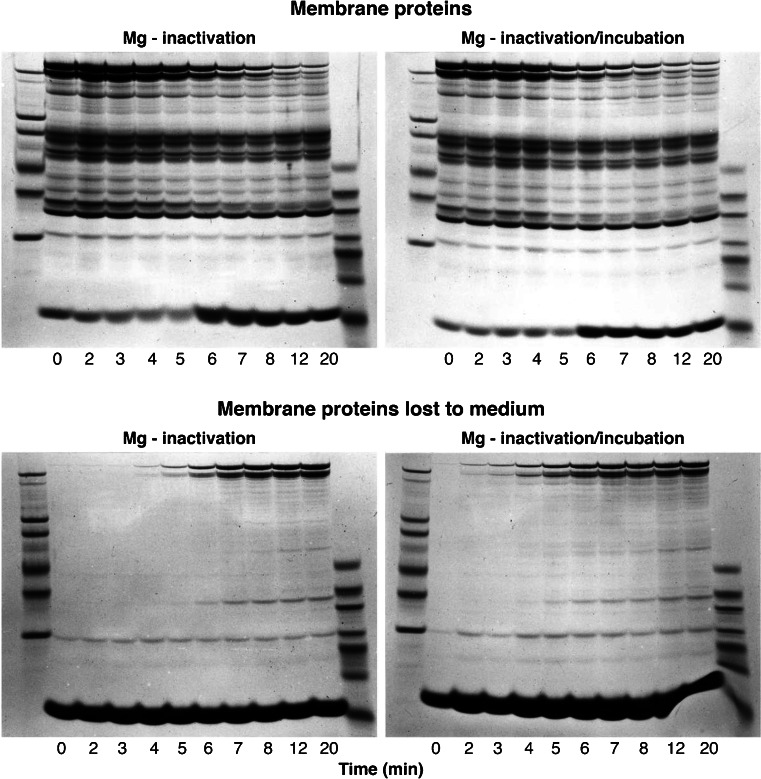
Effect of magnesium ions on the time-dependent patterns of membrane protein changes during spontaneous vesiculation. The experimental protocol was similar to that described in Fig 4. Samples for membrane and supernatant proteins shown on the *left panels* were taken before (*t* = 0) and after switching the temperature of the ghost suspension from 0 to 37 °C, at the times indicated under *each column* (in min). *Left panels* show the patterns obtained from Mg-inactivated samples immediately processed for SDS–gel electrophoresis, as reported in “Methods” (Mg inactivation). *Right panels* show the patterns recovered from duplicate Mg-inactivated samples from each of the originally timed samples after 1 h of incubation at 37 °C before processing for SDS–gel electrophoresis (Mg inactivation/incubation). Extreme *left* and *right columns* in each of the *four panels* are molecular weight standards. *Top panels* show membrane proteins; corresponding *bottom panels* show bands of proteins lost to the supernatant. Notwithstanding imperfect synchronization, it is clear that there is no large-scale spectrin loss (bands 1 and 2, see Fig 3) from membranes to supernatants during the first 4 to 5 min, the most dynamic stages of the spontaneous vesiculation process. Large-scale spectrin loss occurs concurrently with haemoglobin retention in the membrane protein gels (6-min sample) reflecting vesicular sealing. The similitude of the two membrane protein gels (*top panels*), particularly for the samples taken during the first 4 to 5 min of incubation, supports the view that vesiculation arrest by Mg^2+^ results from prevention of spectrin detachment, probably through spectrin cross-linking

Figure 5 shows the results of an experiment similar to that of Fig. 4 in which the changes in membrane and supernatant proteins during spontaneous vesiculation were followed at minute intervals for 8 min to a final 20-min sample. In this experiment, in addition, duplicate Mg-arrested samples were incubated for a further 1 h at 37 °C before SDS–gel processing to investigate whether the observed Mg arrest of vesiculation also prevented spectrin–actin release, as expected if the reversible arrest of all spontaneous dynamic motion was the result of a freeze in cytoskeletal configuration at the instant of interaction with the divalent cation.

The results (Fig. 5, left panels) confirm with greater detail than in Fig. 4 the time-course of spectrin–actin retention and loss and of Hb trapping within sealing vesicles. Full retention of spectrin and actin during the first 4 to 5 min, that is, during the most dynamic stage of the vesiculation process, is particularly noteworthy because it supports the role of the cytoskeletal mesh as the dynamic drive of spontaneous vesiculation. Post-incubation in the Mg-arrest medium (Fig. 5, right panels) reveals a similar pattern of spectrin–actin retention-loss to that of the non-post-incubated samples, the pattern expected from a stabilizing role of Mg ions on the spectrin–actin configuration of the membranes at the instant of Mg exposure. Reversibility of the Mg effect was demonstrated by the continuity of the vesiculation process after washing Mg away and further incubation of the membranes in the lysis medium (not shown). Together with the results in Figs. 4 and 5, the implication is that the configuration of the cytoskeletal mesh at arrest fully recovers its pre-arrest condition after Mg removal.

**Fig. 6:**
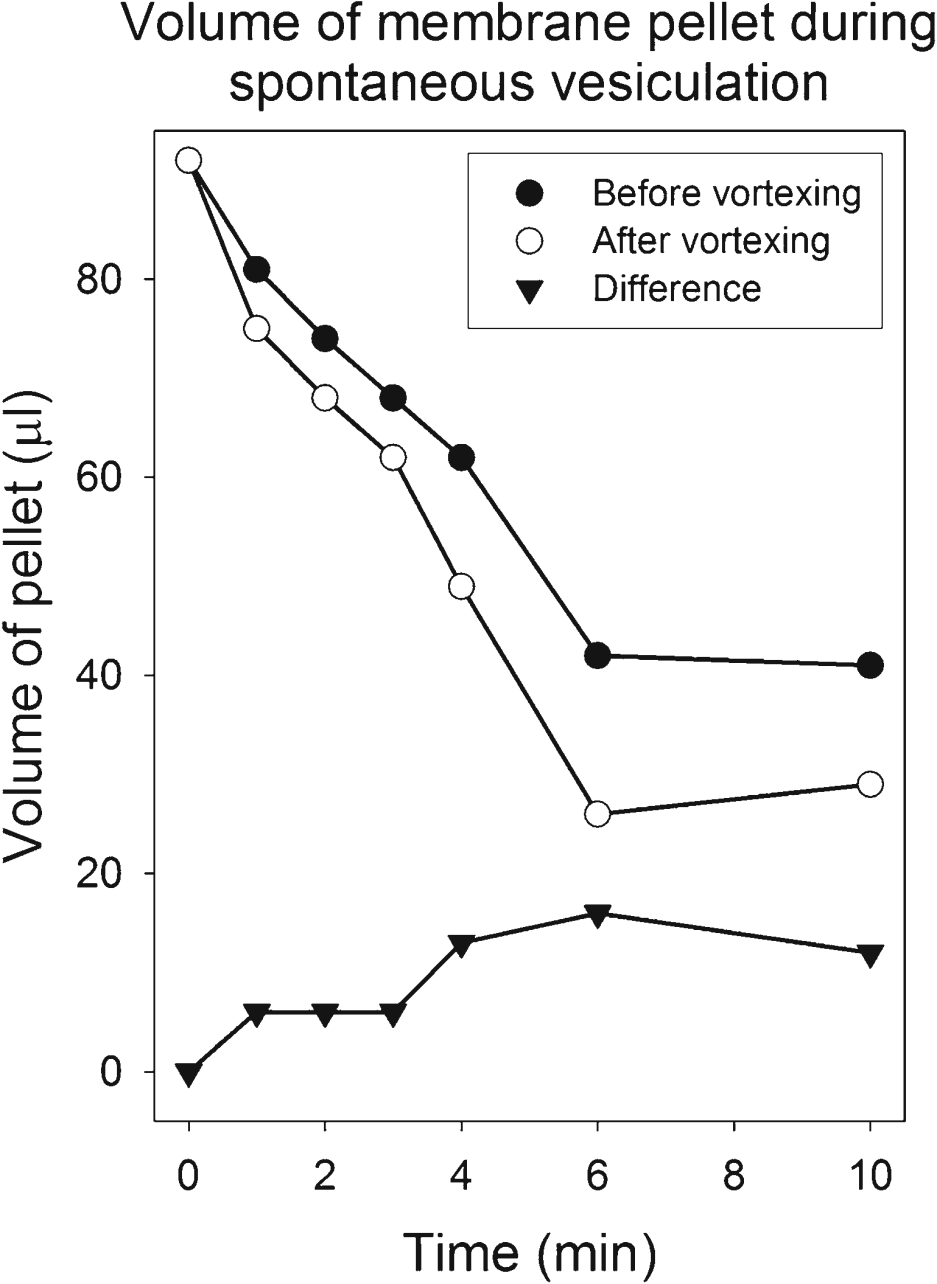
Shear-induced changes in the volume of membrane pellets during spontaneous vesiculation. Red cells were lysed and resuspended at 50 % equivalent haematocrit in L at 0 °C. After 30 min pre-incubation in the ice-bath, vesiculation was initiated (*t* = 0) by transfer to a water bath at 37 °C. Paired Mg-arrested samples were taken at the indicated times. One sample of each pair was vigorously vortexed for 30 s, and both samples were then centrifuged at 12,000×*g* for 30 min at 4 °C, conditions that left no detectable membrane protein presence in the supernatants. The volume of the membrane pellets was estimated from the height of the pellet column on magnified photo records of the microfuge tubes which had been volumetrically calibrated with coloured fluids (the coefficient of variation of the volume estimates was 5.8 %). Results are representative of four similar experiments

We consider next whether or not spectrin–actin detachment during inside-out vesiculation occurs uniformly over the whole membrane area, a question amenable to test by a procedure based on earlier work by Lange and collaborators [22]. Naked membrane patches devoid of spectrin reticulum, if formed during spontaneous inside-out vesiculation, could be revealed by exposing the membranes to intense shearing forces. As shown in Fig. 2, naked membranes deform markedly even under relatively mild convective currents. Exposed to intense shear, they fragment and reseal into tiny vesicles. The volume of membrane pellet (*V*) would be expected to be reduced as the mean equivalent radius (*r*) of the emerging vesicles decreases. At constant membrane area, assuming spherical ghost-to-vesicle conversions, *V* ∼ (*A*/3)*r*. We measured the volume of membrane pellets in Mg-arrested samples throughout spontaneous inside-out vesiculation, in paired samples, with and without vortexing for 30 s. Volume reductions in unvortexed samples reflect mean radius reductions resulting from spontaneous vesiculation; in vortexed samples, they expose additional shear-induced vesiculation on naked membrane patches. The results in Fig. 6 show that the major extent of volume reduction is due to spontaneous vesiculation (74 to 88 % in the four experiments of this series), with a significant and gradually increasing availability of naked membranes patches to additional fragmentation and vesiculation (Fig. 6, triangles).

## Discussion

The results presented here show that, within the bounds of attainable synchronization of spontaneous vesiculation in the ghost population, extensive spectrin–actin detachment and release to supernatant occurs only at the stage when haemoglobin retention exposes vesicular sealing (Figs. 4 and 5). The bulk of the spectrin–actin loss is therefore a terminal event, concurrent with terminal vesicular sealing (Figs. 3, 4 and 5). Further incubation of Mg-arrested samples taken throughout vesiculation does not elicit additional membrane protein loss or altered globin association relative to samples processed immediately (Fig. 5), consistent with a reversible cross-linking effect of Mg ions stabilizing the configuration of the spectrin–actin mesh at the time of Mg exposure.

Electron microscopic images of vesiculating membranes from both right-side out and inside-out ghosts showed time-dependent changes in the appearance of the fibrillar projections lining the inner membrane surface, the morphological correlate of the spectrin–actin mesh [25]. The fibrillar projections changed from an initial uniform distribution to a progressively flaky and patchy appearance representing higher-order spectrin or spectrin–actin aggregates. Some of these aggregates could be seen terminally as fully detached fibres retained within sealed vesicles. The present results (Fig. 6) document progressive formation of naked membrane patches during vesiculation, a correlate to the changing fibrillar morphology. Our data also provides approximate estimates of the relative contributions of active (dependent on spectrin cortex dynamics) and passive (shear-induced on naked membrane patches) components to reductions in mean vesicular radius during spontaneous vesiculation, of about 80 and 20 %, respectively.

In the original molecular model of the spontaneous vesiculation process [25], retention of a spectrin cortex undergoing profound structural changes during the dynamic stages of the vesiculation process was considered necessary for vesicular modelling. The current results confirm large-scale spectrin retention during the membrane dynamic shape changes and large-scale detachment at the terminal vesiculated stage (Figs. 4 and 5). However, the mechanisms by which the structural changes in the spectrin cortex control membrane dynamics remain to be elucidated. Three different mechanisms were proposed in the recent literature to account for membrane dynamics and shape control: (i) localized release of spectrin constraints on the sign and dynamics of the membrane curvature [18, 20, 37], (ii) elastic energy release [1, 8] and (iii) spectrin-oligomerization-driven shape changes [35]. The first two are theoretical models focussed on curling and on egress-associated membrane dynamics in conditions of altered spectrin cortex structure. The third is experimentally based and concerns shape control in intact red blood cells. Nans and collaborators [35] applied cryo-electron tomography to study the topology of the cytoskeletal mesh in intact, unfixed mouse red cells, frozen in physiological buffer. From analysis of the contour length of the spectrin filaments connecting junctional complexes, relative to the fully extended length of the spectrin heterotetramer, they concluded that higher-order oligomers, mostly hexamers and octamers, were prevalent in the mesh network and suggested that spectrin oligomerization dynamics may be central to cell shape control. It is therefore a plausible option that even in the un-physiological conditions in which spontaneous vesiculation occurs, local spectrin oligomerization dynamics, freed from divalent cation stabilizing influences, helps sculpt some of the complex shape transformations of the vesiculating membranes for as long as actin nodal links persist.

A comparative analysis of the CBEV sequences in IOGs and during merozoite egress provides some insights on the likelihood of these alternative mechanisms. In both hypo-osmotic lysis in low-ionic strength, divalent cation-free media and pre-egress host-cell rupture in iso-osmotic, plasma-like media, a large opening is formed in the red cell membrane. In IOGs and after pre-egress rupture, as soon as the hole is formed, the membrane rapidly curls, buckles and everts (the CBE response). But whereas in IOGs curling, buckling and eversion constitute the response of an initially normal-configured cytoskeleton, in the infected cells the CBE response occurs with an extensively remodelled cytoskeleton resulting from pre-egress protease activity [6, 9, 14, 15, 33, 34]. IOGs would never form in the presence of divalent cations or in isotonic, high ionic strength media. CBE prevention by divalent cations in IOGs probably results from spectrin cross-linking, consistent with the results in Fig. 5. On the other hand, egress membranes undergo CBE after rupture in plasma-like media with relatively high Ca^2+^ and Mg^2+^ concentrations. One possible interpretation is that in infected RBCs, pre-egress cytoskeletal remodelling removes the capacity of divalent cations to cross-link spectrin, as if the molecular distances between spectrin strands in the disrupted cytoskeletal mesh became no longer bridgeable by cross-linking action. Indirectly, this line of argument would tend to support constrain release mechanisms for CBE in IOGs and egress because it is hard to envisage similar spectrin oligomerization responses from such different initial cytoskeletal constitutions.

IOGs form instantly upon lysis at 0 °C [25]. The time-courses of CBE in IOGs at 0 °C and of CBEV on egress at 37 °C [1] appear similarly rapid, both completed in a fraction of a second. On the other hand, terminal vesiculation from IOGs takes 5–8 min at 37 °C (Figs. 1, 4 and 5), much longer than the ∼400 ms for post-egress vesiculation. The kinetic differences between the two CBEV sequences are thus essentially confined to the *E*–*V* interval. But the *V* differences between the two processes are more profound. When fluorescent phalloidin A was present in the culture medium during egress, the vesiculated residual red cell membranes appeared strongly labelled with the fluorescent dye indicating substantial retention of the pre-egress cytoskeleton in the post-egress vesiculated membrane [14]. On the other hand, in lysis-generated IOVs, whether IOGs or ROGs, the spectrin cortex has dissociated (Figs. 4 and 5). The vesicles in the post-egress membrane remain aggregated, like bunch of grapes [14], whereas IOVs are mostly free (Figs. 1 and 4), reflecting the persistence (in egress membranes) and loss (in IOVs) of structural linking elements. The few high-speed video-records of egress in which vesiculation can be discerned [1, 10] show vesiculated membranes arising from the everting toroid, as was shown for lysis-formed IOVs, suggesting similar cutting–splicing processes and topologies in the residual vesiculated state, although the inside-out sidedness of the egress-formed vesiculated membrane residue is yet to be established. Whether a common molecular mechanism may account for such profound structural and formation-rate differences between IOVs and post-egress vesicles remains an open question prompting further research.
